# Anxiety and depression levels are different in patients between allergic rhinitis and asthma

**DOI:** 10.1186/1939-4551-8-S1-A52

**Published:** 2015-04-08

**Authors:** Aysenaz Özcan, Adile Berna Dursun, Tugba Cicek

**Affiliations:** 1 Ataturk Chest Diseases and Thoracic Surgery Training and Research Hospital, Turkey; 2 Recep Tayyip Erdogan University School of Medicine, Turkey

## Background

AR and asthma, often seen in younger adults and had social- psychological burden, are common chronic diseases. The objective of the study was to determine and compare the prevalence of anxiety and depression in allergic rhinitis (AR) and asthmatics.

## Methods

Subjects without known psychiatric diseases were consecutively recruited from pulmonary and allergy out-patient clinics at the third level hospitals. Diagnosis of asthma and AR was based on GINA and ARIA guideline, respectively. Only patients without asthma were chosen for AR group in order to compare with asthma. Depression and anxiety symptoms were evaluated using Beck Depression Inventory (BDI) and Beck Anxiety Inventory (BAI).

## Results

Study group consisted of 60 patients-30 with asthma and 30 with AR. All patients had moderate-to severe diseases. AR group was younger; but the mean disease duration was not significantly statistically different. AR group had higher education history than those of asthmatics. Number of smokers in both groups were equal. Female gender was predominant in both groups. In asthmatics, 53.3% had positive skin prick test with aeroallergens. Polysensitization rate was 56.25% and 63.3% in asthmatics and AR group, respectively. The most common sensitization pattern was mite plus mould sensitization. The mean BDI and BAI scores were 11.47±11.14 and 15.22±11.31 in the whole group. Both BDI and BAI scores were significantly statistically different in the study groups (Table 1). Asthma and low level of education was found as the important factors for BAI in multiple regression analysis.

**Table 1:** Beck Depression Index and Beck Anxiety Index in the study population

	Asthma n=30	Allergic rhinitis n=30	p
BDI, mean±SD	15.63±12.61	7.3±5.6	.006

BAI, mean±SD	21.23±11.74	9.2±6.9	.000

## Conclusions

AR patients without asthma seem to have better coping mechanism than that of asthmatics.

